# Convolutional-Neural Network-Based Image Crowd Counting: Review, Categorization, Analysis, and Performance Evaluation

**DOI:** 10.3390/s20010043

**Published:** 2019-12-19

**Authors:** Naveed Ilyas, Ahsan Shahzad, Kiseon Kim

**Affiliations:** 1 School of Electrical Engineering and Computer Science, Gwangju Institute of Science and Technology (GIST), Gwangju 61005, Korea; naveedilyaas@gmail.com; 2 Department of Computer and Software Engineering (DCSE), College of Electrical and Mechanical Engineering (EME), National University of Sciences and Technology (NUST), Islamabad 44000, Pakistan; ahsan.shahzad@ceme.nust.edu.pk

**Keywords:** deep learning, crowd analysis, smart cities

## Abstract

Traditional handcrafted crowd-counting techniques in an image are currently transformed via machine-learning and artificial-intelligence techniques into intelligent crowd-counting techniques. This paradigm shift offers many advanced features in terms of adaptive monitoring and the control of dynamic crowd gatherings. Adaptive monitoring, identification/recognition, and the management of diverse crowd gatherings can improve many crowd-management-related tasks in terms of efficiency, capacity, reliability, and safety. Despite many challenges, such as occlusion, clutter, and irregular object distribution and nonuniform object scale, convolutional neural networks are a promising technology for intelligent image crowd counting and analysis. In this article, we review, categorize, analyze (limitations and distinctive features), and provide a detailed performance evaluation of the latest convolutional-neural-network-based crowd-counting techniques. We also highlight the potential applications of convolutional-neural-network-based crowd-counting techniques. Finally, we conclude this article by presenting our key observations, providing strong foundation for future research directions while designing convolutional-neural-network-based crowd-counting techniques. Further, the article discusses new advancements toward understanding crowd counting in smart cities using the Internet of Things (IoT).

## 1. Introduction

Crowd counting (CC) aims to count the number of objects, such as people, cars, cells, and drones in still images or videos. It can be performed in different ways, such as digital-image processing, machine learning, and deep learning. More specifically, crowd counting can be done through various state-of-the-art techniques, such as counting by detection [1,2,3] regression [4,5,6,7,8], density estimation [9,10] and clustering [11,12,13,14]. The problem of crowd counting is of significant importance in computer vision due to its wide variety of applications in urban planning, anomaly detection, video supervenience, public safety management, defence, healthcare, and disaster management [15,16,17].

Crowd-counting techniques face many challenges, such as high cluttering, varying illumination, varying object density, severe occlusion, and scale variation caused by different perspectives [18,19,20,21,22]. For instance, high cluttering can distort the resolution of an estimated map, and light illumination can reduce its accuracy. Further, varying object density reduces prediction accuracy due to nonuniform density distribution. Similarly, severe occlusion increases prediction error, and scale variation reduces both counting prediction and density-map resolution.

Due to a wide variety of applications, from commercial to military purposes, with significant importance in computer vision, crowd counting is a challenging scientific problem to be solved. A number of researchers tried to provide detailed surveys and analyses of previous techniques by considering various crowd features. These traditional crowd-counting techniques mainly focus on handcrafted low-level crowd features. These low-level features are selected, extracted, and transformed into an organized input for the regression model that is used for loss-function evaluation and minimization. In this regard, comprehensive analysis was provided by Zhan et al. [23] for general crowd counting. They mainly reviewed vision and nonvision problems. In vision-based problems, crowd modelling is based on extracted information from visual data and employed for crowd-event inference. Nonvision approaches, on the other hand, aim to describe and predict the collected effects of crowd behavior by rectifying the relationship between features. Later on, Zitouni et al. [24] focused on crowd-counting models with emphasis on their limitations. Their main contribution was the categorization of crowd-modelling techniques into motion-flow-based models, learnt-appearance-based models, and hybrid approaches. Motion-flow-based models were further subcategorized into optical-flow-based models, Lagrange based methods, and background-subtraction-based models. The authors in [25] investigated crowd-counting techniques by considering different categories, like holistic, intermediate, and local approaches. The authors in [26] focused on conventional and convolutional-neural-network (CNN)-based single-image crowd-counting techniques. They mainly compared the properties of handcrafted crowd-counting techniques with CNN-CC techniques.

To sum up, the existing above-mentioned surveys and analysis, except for [26], mostly focused on conventional approaches that emphasised handcrafted features to improve the accuracy of crowd analysis. In [26], traditional and CNN-CC algorithms were reviewed. However, the authors did not review recent CNN-based crowd-counting algorithms, which are evaluated on the most challenging and multivariant datasets [27,28]. Further, the aforementioned works did not analyze the advantages and limitations of each technique. The limited categorization of CNN-CC techniques restricts future researchers from fully understanding the scope and available room for improvement in any category. Finally, they did not conduct a quantitative comparison in terms of prediction accuracy. These drawbacks/deficiencies in existing works indirectly and negatively impact system performance. For instance, due to a lack of categorization, the whole domain under any specific category has not been explored. Not considering key aspects such as advantages, disadvantages, limitations, intrinsic features, and multivariant datasets meant that in-depth properties are ignored in the design process of a crowd-counting algorithm. Such scanty investigations usually lead to a huge diversity of simulation results in comparison to the real crowd count.

Motivated by the above-mentioned deficiencies in previous surveys [23,24,25,26], we comprehensively reviewed the most recent CNN-CC techniques to understand the newest trends and highlight room for future research in any particular area. Understanding crowd-mobility behaviors would be a key enabler for crowd management in smart cities, benefiting various sectors such as public safety, tourism, and transportation. The main theme of crowd-counting categorization is to help researchers to further exploit and dive deep into any particular branch to obtain maximum output. This article discusses existing challenges and recent advances to overcome them and allow the sharing of information across stakeholders of crowd management through Internet of Things (IoT) technologies. To summarize, this paper makes the following contributions.

- We specifically reviewed recent CNN-CC techniques in order to highlight deficiencies, advantages, disadvantages, and key features in each category.
- We categorized CNN-based methods into three main categories to fully understand evolving research aspects. Previously, authors in [26] categorized CNN-based approaches into two main categories (network-based and training-approach-based). However, by reviewing the literature and observing the overall crowd-counting mechanism from different perspectives, we realized the need for a new category, and thus introduced image-view-based methods.
- Image-view-based CNN-CC techniques (Image-view-CNN-CC) were further subdivided into arial-view-based (camera and object are perpendicular to each other) and perspective-view-based (camera and object are parallel to each other) methods. Due to this inclusion, crowd counting in health care (microscopic images), counting through unmanned aerial vehicles (UAVs) is further investigated under arial-view-based methods. Moreover, scale-varying issues caused by different perspectives can be further investigated in detail under perspective-view-based methods.
- We provide detailed quantitative comparison (in term of n Mean Absolute Error (nMAE) within each subcategory of the three main categories, and overall performance-based conclusion under different datasets, such as UCF, WE, STA, and STB.
- By observing different aspects of CNN-CC, we also highlighted the features of each subcategory with quantitative comparison that provides a strong foundation for future research in highly diverse and robust scenarios.

The rest of paper is organized as follows. Section 2 is focused on traditional crowd-counting methods and image analysis. In Section 3, we discuss the complete and detailed operation of the crowd-counting mechanism. Section 4 is focused on the categorization of CNN-CC techniques by considering their features, datasets, and architectures. In Section 5, we discuss applications of CNN-CC techniques. In Section 6, we discuss the implications of 3D crowd counting. In Section 7, we provide a quantitative comparison between different CNN-CC techniques. Finally, Section 8 provides the conclusion with future research directions.

## 2. Traditional Crowd Counting and Image Analysis

### 2.1. Crowd Counting

**Figure 1:**
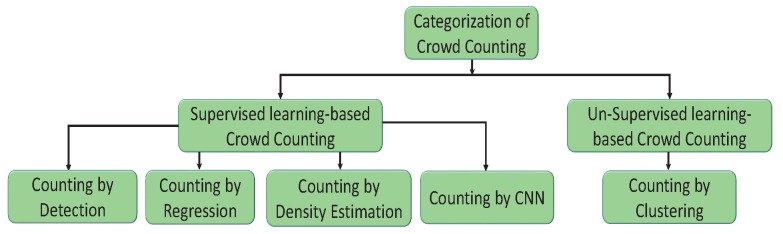
Categorization of crowd-counting techniques.

A crowd is defined as a large gathering of people for a specific reason, such as religious occasions, sports events, and political gatherings. Crowd counting is defined as estimating or counting the number of people in an image or video [29,30]. Techniques for crowd counting are divided into two basic categories: supervised and unsupervised crowd counting. In supervised crowd counting, the input data are known and labelled, and the machine is only used to determine the objective function (hidden pattern). In unsupervised crowd counting, the used data and labels are unknown, and the machine is used to categorize and label the raw data before determining the objective function. These categories are further divided into different types, as shown in Figure 1. Supervised crowd counting is further divided into counting by regression, density estimation, detection, and CNN [31,32]. The unsupervised category, on the other hand, includes counting by clustering. Their descriptions are as follows.

#### 2.1.1. Counting by Detection

Counting by detection can be defined as a method to compute the abstraction of image information and local decisions at every point to know about features of a particular type at that point. The authors in [33] proposed a CNN-based hybrid hidden Markov model (HHMM) for speech recognition. The HMM is used to obtain inherent dynamic features that can be used for anomaly detection in crowd analysis. The authors in [34,35] found a solution for reconstructing full-body locomotion that could be used in 3D crowd analysis and abnormal-behavior detection. The earlier research focused on detection-based counting to count the number of people in a scene [3]. Through a sliding-window detector, detection could be monolithic or part-based. Traditional pedestrian-detection techniques used monolithic detection [16,36,37,38]. In these techniques, a classifier is trained by using different features, including Histogram Oriented Gradient (HOG) [16], edgelet [39] and a shapelet [40] extracted from the body of people. The monolithic way of detection performs very well in low-density crowds, but its performance degrades in high density. Therefore, researchers were motivated to address this issue by using part-based detection techniques [41,42] that use boosted classifiers for specific body parts, including shoulders and head, to estimate the count in that area [43].

#### 2.1.2. Counting by Regression

Counting by regression is carried out to obtain a more robust and accurate function via known inputs of images and output (ground truth). The authors in [34,35] determined a solution on the basis of reconstructing full-body locomotion that could be applicable in 3D crowd analysis and anomaly detection. Regression-based crowd-density estimation was first exploited by Davies et al. [7]. The extraction of low-level features (foreground area and edge features) is carried out in the video frame. The total edge count and foreground area are extracted from the raw features. In this way, a linear-regression model was developed to establish mapping between actual and estimated count. Shape- and part-based detectors are not successful in the presence of high-density crowds and high-clutter backgrounds. The main components that establish counting by a regression pipeline are low-level feature extraction and regression modelling [4]. Different features, such as gradient, foreground, and edge features, and textures are used to encode low-level information. Further, standard background-subtraction techniques are used for the extraction of foreground features that are removed from foreground segments. Blob-based holistic features, such as perimeter, area, and perimeter–area ratio have had promising results [4,25]. However, these techniques focus on the global properties of the scene. Local features and textures like Gray Level Co-Occurence Metrics (GLCM), HOG, and Local Binary Pattern (LBP), are used to further improve the accuracy of classification, detection, and crowd counting. After the extraction of local and global features, a variety of regression methods, including linear [44], Gaussian, [45], and ridge regression [7], and NNs [46] are used to learn mapping between the actual crowd count and low-level features.

#### 2.1.3. Counting by Density Estimation

Counting through density estimation is employed to obtain an estimate by using observed data of an unobservable probability-density function. This technique has made it possible to overcome the problem of occlusion and clutter by using spatial information with a density-estimation approach. For example, Lempitsky et al. [10] incorporated spatial information by proposing linear mapping between local features and estimated-density (ED) maps. The difficult task of detecting and localizing individual objects has been eliminated by calculating image density whose integral in any particular region provides the estimated count of that region. In [10], cutting-plane optimization is used to solve convex optimization tasks by introducing a risk-based quadratic cost function.

#### 2.1.4. Counting by CNN

Though detection, regression, clustering, and density-estimation-based crowd-counting techniques perform well to some extent by using handcrafted features, for crowd analysis, motion analysis, and the 3D construction of body parts, different types of CNN- and LSTM-based algorithms have been proposed. In particular, the authors in [47] and [48] proposed a CNN-based descriptor and LSTM-based network to obtain motion and appearance information along the tracks of human body parts. Similarly, the authors in [49] investigated 3D face-model construction by using a 2D view of the face. Further, the authors in [50] investigated deep-learning architecture for the classification of a driver’s actions. Abstractive text summary using a generative adversarial network was done by the authors in [51], while the authors in [52] proposed a CNN-based technique to obtain high representational features for the detection of secondary protein structures. In order to further improve accuracy, researchers used CNN-based crowd-counting techniques [21,53,54]. Counting through CNN employs convolution, pooling, Rectified Linear Unit (RelU), and Fully Connected Layers (FCLs) to extract features that are used to obtain the density map [55]. Counting through CNN is more efficient in terms of accuracy, but at the cost of high computational complexity.

#### 2.1.5. Counting by Clustering

Counting by clustering relies on the assumption that visual features and individual motion fields are uniform, so similar features are grouped into different categories. For example, [13] used a Kanade–Lucas–Tomasi (KLT) tracker to obtain low-level features, and then employed Bayesian clustering [14] to find the approximate number of people in an image. The aforementioned methods explicitly model appearance features. Thus, false estimation arises when people remain in static position or when objects repeatedly share the same trajectories. Hence, we concluded that counting by clustering performs better in continuous image frames.

### 2.2. Image Analysis

Image analysis is widely used to extract useful information from an image, specifically digital images, by using different techniques like digital-image processing, machine learning, and deep learning [56]. Inspired by the phenomenon of the human-visual-cortex system, CNNs extract high-level features from an image. Image analysis has more subfields like pattern recognition, digital geometry, medical imaging, and computer vision [57,58,59,60]. These subfields cover various modern-day applications in astronomy, defence, filtering, microscopy, remote sensing, robotics, and machine vision [61,62].

### 2.3. Unique Challenges of CNN-Based Image Crowd Counting

**Figure 2:**
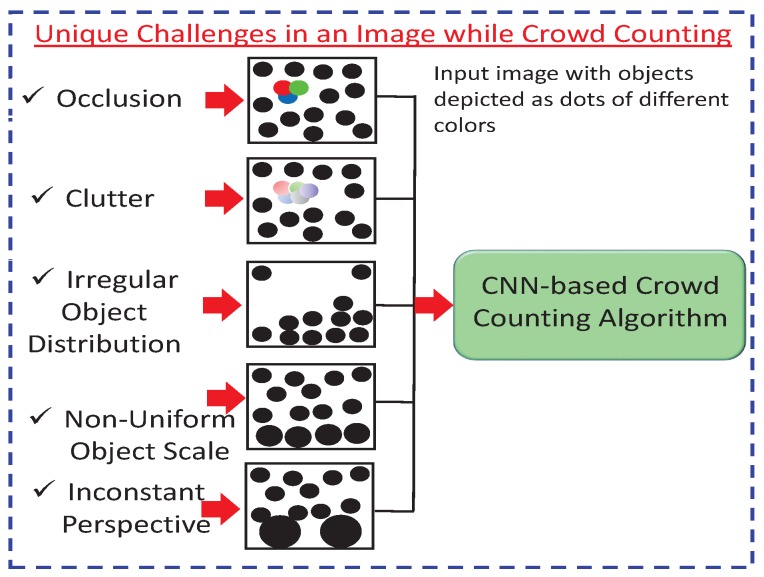
Unique challenges of convolutional-neural-network (CNN) crowd counting (CC) techniques in an image.

CNN-based crowd counting faces many challenges that restrict the counting accuracy of these networks (i.e., MAE, MSE, and ED) and the resolution of the density map. These challenges are depicted in Figure 2 and explained below.

- Occlusion occurs when two or more objects come very close to each other and merge, so that it is hard to recognize individual objects. Thus, crowd-counting accuracy is decreased [18].
- Clutter is a kind of nonuniform arrangement of objects that are close to each other. It is also related to image noise, making recognition and counting tasks more challenging [19].
- Irregular object distribution refers to varying density distribution in an image or a video. For irregular objects, counting through detection is only viable in sparse areas. On the other hand, counting by regression overestimates the sparse areas and is only viable in dense areas. Thus, the irregular distribution of an object is a challenging task for crowd counting [20].
- Nonuniform object scales often occur due to different perspectives. In counting, objects close to the camera look larger when compared to ones farther away. The nearest objects have more pixels than far-away objects. Thus, ground-truth and actual-density estimations are affected by the nonuniform pixel distribution of the same object [21].
- An inconstant perspective occurs due to different camera angles, tilt, and the up–down movement of the camera position. Object recognition and counting accuracy are greatly affected by varying perspectives [22].

### 2.4. Motivation for Employing CNN-Based Image Crowd Counting

Traditional handcrafted crowd-counting techniques such as those in [1,14] perform well if the training dataset has a low computational cost. However, challenges like occlusion, clutter, and scale variation reduce the accuracy of such traditional methods. In addition, the ED map obtained by employing these handcrafted methods has a low resolution that limits their applicability in many areas, such as medical imaging and military applications. In short, the manual nature of feature extraction by handcrafted methods makes them less (non)adaptive to evolving crowd-counting demands. By observing the above-mentioned deficiencies in traditional crowd-counting algorithms, and the success of CNNs in numerous computer-vision applications, researchers were inspired to exploit their ability in estimating the nonlinear feature density maps of crowd images [53,54,55]. These density maps can be utilized in machine-learning processes for more accurate prediction/estimation of the crowd count [63,64]. Further, up- and downsampling, scale aggregation, and preclassification with a multicolumn approach could also be used to increase the accuracy of crowd counting. On the other hand, deconvolution [65] and Generative Adversarial Networks (GANs) [66] can be employed to enhance the quality of a density map for medical applications.

## 3. CNN-Based Crowd Counting: Overview

**Figure 3:**
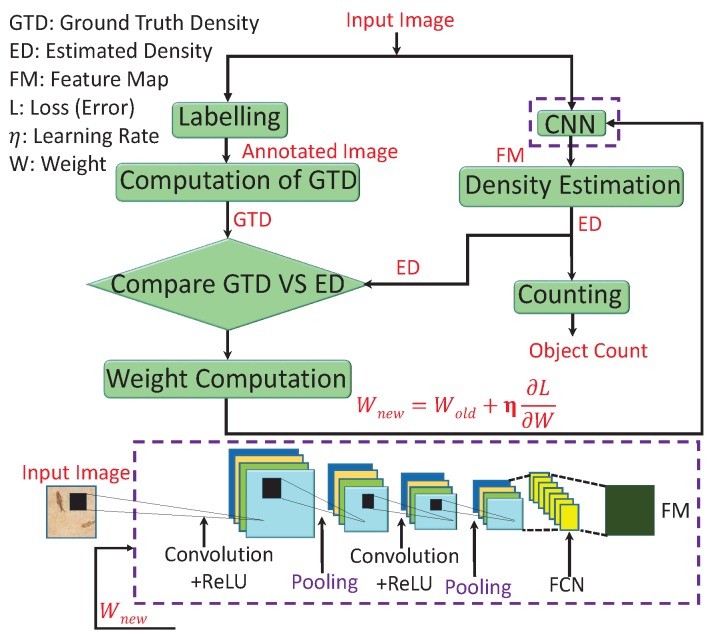
General form of CNN-CC algorithm. Crowd-counting mechanism starts from object annotation in an image to density estimation; object counting is depicted. General framework of crowd counting (**top**), and CNN working is expanded (**bottom**).

CNNs are useful in numerous applications, such as signal processing, image processing, and computer vision. In this regard, various CNN-CC algorithms were proposed to cope with major issues like occlusion, low visibility, inter- and intraobject variation, and scale variation due to different perspectives. A generic CNN-CC flow diagram is shown in Figure 3 that depicts two approaches. The first, on the left, found ground-truth density (GTD) except for the last two blocks, which were used for comparison and error computation. The second, on the right, computed ED and crowd counting. The description of each block is as follows.

*Labelling*: In machine learning, annotation is a process of labeling data such as text, audio, and image. The annotated data are used by a computer or computers to recognize similar patterns in unseen data. There are different annotation categories, such as bounding-box annotation, polygonal segmentation, line annotation, landmark annotation, 3D cuboids, and dot annotation [67]. These different types of annotations are used to obtain the ground truth. In crowd counting, dot annotation is the first step to compute the GTD, and it is carried through various scientific tools like Labelbox, LabelMe, and RectLabel [10].

*GTD computation*: Ground truth can be defined as the information provided by direct observation instead of inference. There are different ways to obtain the GTD, such as Gaussian kernel, geometry-adaptive kernel, and GANs [8]. The geometry-adaptive kernel performs better than the Gaussian kernel. This is due considering spread-factor-based geometric information. Further, a combination of generative and discriminative networks brings the generated image very close to the original one. Therefore, the obtained GTD from GANs has better quality as compared to that of the Gaussian, geometry, or body-aware techniques.

*GTD and ED comparison*: In crowd counting, ED and GTD are compared to compute the loss between estimated output and ground truth. In the literature, different techniques were employed to compute loss, such as cross-entropy and MSE [17]. A combination of sigmoid and MSE converge much slower as compared to sigmoid and cross-entropy due to the gradient-vanishing problem. Cross-entropy, on the other hand, performs well on classification problems, but better performance was shown in terms of MSE in regression-based problems.

*Weight Computation*: After comparing the loss between ED and GTD, the next step is to update the network weight to minimize loss. The updated weight is computed by $W_{new}=W_{old}+\eta \frac{\partial L}{\partial W}$. This weight update process (backpropagated to the CNN) is terminated when loss is minimized (process converges). $W_{old}$ and $W_{new}$ depict the old and new weights, where the last term is the combination of learning rate η and change in loss with respect to weight [17].

*CNN*: In CNN-CC, the image is first fed into the CNN that consists of convolution, ReLU, pooling, and FCL, as depicted in Figure 3 (bottom). The CNN functions by extracting image features in the form of a feature map. These features are fed into the regression model for estimating the density map for crowd counting. CNN can be categorized into single, multi-, and single with scale-aware networks. Depending on the application, the complexity (single–multi column) and layers of CNN can be optimized to obtain the desired results. These categories are further optimized to provide strong and granular-level foundation for researchers in the future.

*Density estimation*: It can be defined as a way to estimate the probability density function of a random variable on the basis of observed (ground-truth) data. There are different ways to obtain the ED of a crowd, like density estimation by clustering, detection, and regression [3,7,13,68]. Detection-based techniques perform well with sparse crowds, while regression-based methods perform well on dense-crowd environment, and they overestimate crowds in sparse patches. A combination of detection and regression can be used to achieve better performance in both sparse and dense scenarios.

*Counting*: It is a method that is performed after the computation of a density map to count the number of objects (people, cells, cars, etc.) in an image or video. Different well-known handcrafted techniques perform according to image density [10]. For example, in sparse-density images, counting by detection performs well due to a lack of overlapping objects, while CNN-based methods perform well on images with a diverse density range.

Unique challenges faced by CNN-CC algorithms include a complex network architecture, increased number of parameters, high computational cost, and real-time deployment. Traditional handcrafted crowd-counting algorithms can be deployed for real-time monitoring at the cost of reduced accuracy and a low-resolution density map. These techniques also fail to obtain the desired results in high occlusion, a diverse density range, and scale-varied environments. On the other hand, CNN-CC algorithms perform better in terms of prediction accuracy and resolution. Traditional handcrafted methods have less computational cost. The majority of applications aim for high prediction accuracy. Many researchers tried and succeeded to minimize complexity. Hence, growing trends towards CNN-CC techniques motivated us to review and analyze the latest and most well-known research articles on the most challenging datasets.

## 4. Categorization of CNN-CC Techniques

The categorization of CNN-CC techniques plays an important role in their understanding at a granular level. Such a level of understanding enables researchers to design distributed control and monitoring algorithms for various crowd-counting applications in military combat, disaster management, public gatherings, etc.

**Figure 4:**
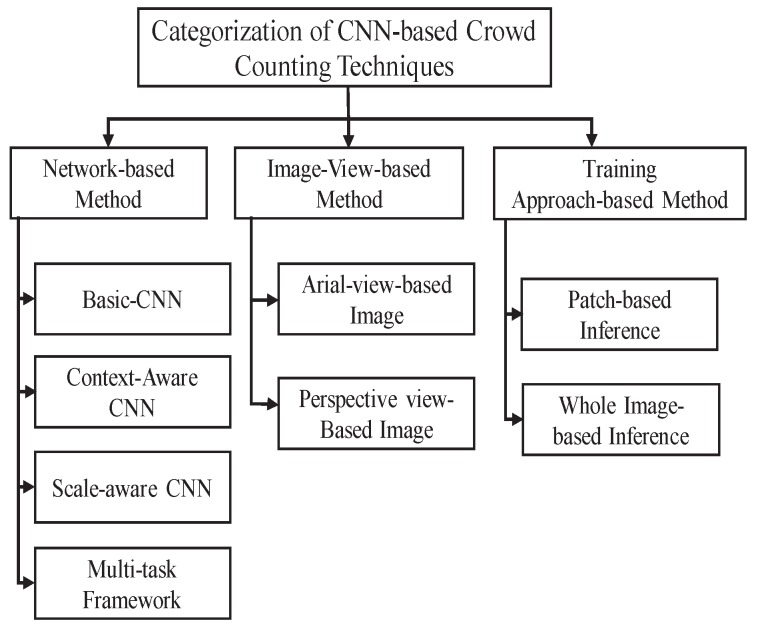
Categorization of CNN-CC techniques.

The only CNN-CC categorization done by Sindagi et al. [26] conducted a very limited research survey of 17 research articles in two main categories, with 17% of articles from 2015, 64% from 2016, and only 17% from 2017. They categorized existing CNN-based techniques only on the basis of network properties and training sets. In order to cover new research articles with evolving architectures and future requirements in terms of datasets and algorithm design, we contribute by adding a new category of CNN-CC techniques, as shown in Figure 4. Inclusion of the third category based on the orientation of input image plays a significant role in the design of CNN-CC architectures and algorithms by understanding the dynamics of the input image. Moreover, we cover 52 of the latest research articles in three main categories, with only 5.76% research articles from 2015, 23.07% from 2016, 25% 2017, and 46.15% from 2018.

Since datasets, in terms of their intrinsic features, play a vital role in the design of CNN-CC algorithms, we provide a brief description of the available datasets prior to categorization details of the CNN-CC techniques. Currently available datasets are of two types, public and private. Public datasets are those publicly available on the Internet, and private ones are the intellectual property of their corresponding authors/organizations. We list five of the most well-known and popular datasets, and their intrinsic features in Table 1.

### 4.1. Network-CNN-CC Techniques

**Figure 5:**
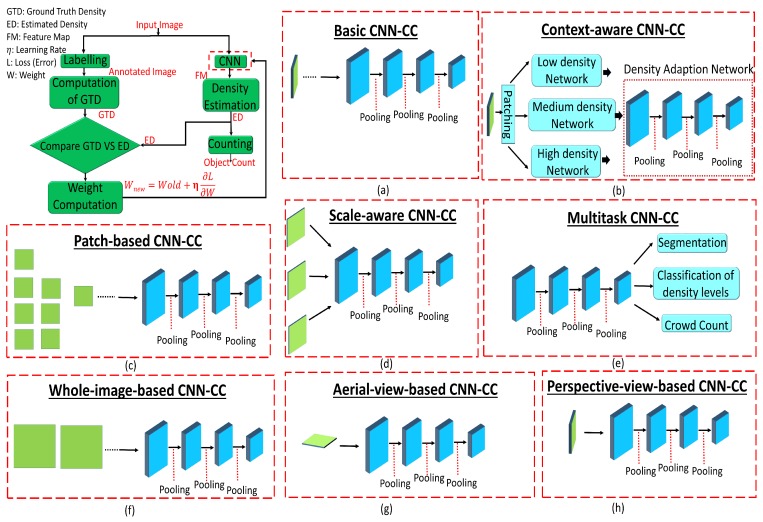
Architectures of different subcategories: (**a**) basic-CNN-CC, (**b**) context-aware CNN-CC techniques (context-CNN-CC), (**c**) patch-based-CNN-CC, (**d**) scale-aware CNN-CC techniques (scale-CNN-CC), (**e**) multitask-CNN-CC, (**f**) whole-image-CNN-CC, (**g**) aerial-view-CNN-CC, and (**h**) perspective-CNN-CC.

Techniques in which the network is modified in terms of layers or columns, and the inclusion of any other module for classification, segmentations, and surveillance ultimately changes the properties of the actual network are called Network-CNN-CC. Techniques under this category are very useful for obtaining high-level crowd features that may lead to significant improvement in a diverse range of densities, such as religious and political gatherings, and sports events. Although techniques in this category play a vital role in obtaining contextual information with varying scales, due to a complex architecture, these types of techniques may not be computationally suitable for real-time crowd counting. Further, Network-CNN-CC-based techniques are subcategorized into basic-CNN-CC, context-CNN-CC, scale-CNN-CC, and multitask-CNN-CC, as shown in Figure 5. Their details are as follows.

#### 4.1.1. Basic-CNN-CC Techniques

Crowd-counting techniques that have a basic CNN architecture are in this subcategory. Basic-CNN-CC techniques can be regarded as the pioneer of deep-learning methods for density estimation that can be applied to obtain a crowd count in real time due to the simple network architecture. Table 2 shows Basic-CNN-CC with their features, used datasets, and architectures.

Fu et al. [70] proposed a bilevel density-estimation method by using a basic CNN architecture. Their first task was to estimate crowd density (i.e., to extract crowd features of different density levels). Estimation speed is increased by removing similar connections. Their second task was to classify discriminative features by using a cascaded classifier. Similarly, a residual learning method with an inception-layer-based technique was proposed in [71] to count the number of cars by dividing an image area into overlapped patches. A stride was adjusted to distinguish nonlocalized cars with contextual information in order to reduce the MSE. Wang et al. [72] proposed an FCNN model with argumentation strategy to increase the number of training data for more robustness of dense and diverse environments. Zhao et al. [73] proposed a CNN model to count the number of people crossing a line in surveillance videos. The original problem was divided into two subproblems (estimation of crowd density and crowd velocity) for reducing the complexity of the main problem. In [74], the authors proposed a deep-learning approach to estimate mid- and high-level crowds in an image. A regressor was used to estimate the number of individuals in a local area, while its overall density was estimated by adding the estimated densities of local regions. In their work, a feature vector was learned by using ConvNets architecture for estimating crowds in their respective local regions. The authors in [75] used a basic CNN for multiple applications that included indoor and outdoor counting. Layer boosting (i.e., increasing the number of trained network layers to iteratively train a new classifier that is used to fix the errors of the previous one) and selective sampling (i.e., minimizing the impact of low-quality samples) are used to reduce processing time and enhance counting accuracy. Four ensemble networks are fine-tuned by training every network on the basis of previous errors.

*Remarks*: Most techniques under this subcategory mainly focus on density estimation instead of crowd count. These techniques may not perform well in highly occluded and varying perspective scenarios due to an oversimplified architecture. In these techniques, the speed of density estimation can be enhanced by removing redundant samples. By iteratively reducing errors in different network layers, error-rate probability can also be reduced.

#### 4.1.2. Context-CNN-CC Techniques

Crowd-counting techniques that utilize both the local and global contextual information of an image for improving counting accuracy fall into this subcategory. The contextual information of an image means a relationship of nearby pixels (i.e., neighboring information) with a targeted area for overall improvement. Techniques under this category are very useful in applications that need contextual information, such as counting the number of moving drones or the number of cars in parking lots. These techniques are also helpful for obtaining density level and distribution in various density-based images. Table 3 shows context-CNN-CC with their features, used datasets, and architectures.

For instance, the idea of an every-day object count was proposed by the authors in [76] by considering the novel idea of associative subitizing (humans’ ability to give quick count estimates/assessments for small object counts). Zhang et al. [77] proposed an attention model to detect head location (high probability indicates head location). Similarly, multiscale feature branches were used to suppress the nonhead region. Li et al. [78] used a combination of CNN and dilated convolution (expanded kernels to replace pooling) for improving the quality of a density map. A dilated convolutional layer was also used for combining contextual information in diverse congested scenarios. Han et al. [79] proposed a CNN–Markov random field for crowd counting in still images. They divided the whole image into small overlapping patches, so that features were extracted from the overlapping patches, and fully connected NNs were used to regress the patch count. The adjacent patches had high correlation due to overlapping. Correlation was used by MRF to smooth people counts within adjacent local patches to improve the overall accuracy of the crowd count. In [80], the authors proposed a density-adaptation-based network to accurately count the number of objects. A generalized framework was proposed that was trained on one dataset and then adapted on another. Density level was computed by selecting a network that was trained on different datasets. The architecture consisted of three networks: a density-adaptation network that was used to identify low or high density, and the other two networks were responsible for counting. Liu et al. [81] proposed a deep recurrent spatially aware network in which a spatial-transformer module was used for counting while simultaneously tackling both scale and rotation variations.

**Remark** **1.** *Real-time contextual information can be employed by using dilated convolution. More specifically, a deeper dilated CNN can be used to enhance the quality of density maps, and an adaptive density network can be used to enhance counting accuracy. However, such contextual information is obtained at the cost of higher network complexity. As a result, techniques in this subcategory may not be feasible for real-time applications with low complexity demands/requirements.*

#### 4.1.3. Scale-CNN-CC Techniques

Basic-CNN-CC techniques that have evolved in terms of scale variations (for robustness and accuracy improvements) are called Scale-CNN-CC techniques. Scale variation means varying the resolution caused by different perspectives. The techniques in this category play a vital role in enhancing accuracy in highly congested and occluded scenarios. The extraction of multiscale patches from an input image makes the goal comparatively easier for crowd counting. This may increase accuracy in a dense and diverse range of datasets such as UCF and STA. However, these techniques rely on the extraction of multiscale patches with a complex architecture. Table 4 depicts the limitations and merits of different scale-CNN-CC methods. The negative sampling and data-driven approach is missing in all the listed methods.

Liu et al. [82] proposed a geometric-aware crowd-density-estimation technique. An explicit model was proposed to deal with perspective distortion effects. Huang et al. [83] reduced the computational cost by investigating the idea of stacked pooling. Instead of using multiscale kernel pooling, stacked pooling is used to extract scale information for making it applicable in real-time applications. Later on, Kang et al. [84] used an image pyramid to deal with scale-varying issues in an image. Instead of changing the filter size, feeding downsampled images into the network was efficient for crowd-counting accuracy. Then, predictions from different scales were fused to obtain the final ED. The authors in [85] proposed a combination of a shallow and a deep network to effectively capture high-level semantics (face, body) and low-level features to accurately estimate crowd density in scale-varying conditions. The authors in [86] proposed a single column multiscale cost effective method for real-time applications. By using a single column with a multiscale blob, scale-related features were extracted. These scale-related features generated by the network were used for dense crowd counting. In [87], the authors proposed a combination of gating (multiclass classifier) and a multiple-expert CNN. The gating CNN automatically directs the input patch to the expert CNN that makes the algorithm robust for large appearance changes. Onro-Rubio et al. [88] proposed two methods to address crowd appearance and scale variation in an image. First, a counting CNN was proposed to map image appearance into its density map. Second, a congested and varying scale region is tackled through the Hydra CNN without any geometric information. The Hydra CNN used the pyramid of patches extracted from multiple scales for density estimation. The authors in [89] proposed a multiscale multitask crowd-counting algorithm with an aggregated feature vector. Multiscale features were basically combined into a single vector, a ‘vector of locally aggregated descriptor’, which was optimized by backprorogation. Moreover, a data-argumentation approach was used to increase the size of the training data. Cao et al. [90] proposed an encoder–decoder-based CNN to reduce computational complexity. By avoiding a multicolumn CNN with a classifier, a simpler scale-aware network (SANet) was used to address scale-varying issues. Further, transpose convolution was used to enhance the quality of the density map. Motivated by the success of GANs in an image for image-translation problems, the authors in [91] employed GANs for crowd counting. The GANs were used for the translation of the image and its patches into generated maps. The actual GTD was compared with the generated map to find the best-resolution density map (high-quality). A novel regularizer adversarial cross-scale consistency pursuit network (ACSCP) was proposed to maintain the parent (whole image) and child (four patches) relationship for reducing counting loss (previously caused by averaging). By using adversarial loss, the distance between the parent density map and the concatenated-image density map was calculated for minimizing loss. This regularizer performed well as compared to $l_{2}$ regression loss.

**Remark** **2.** *A greater pooling range (multikernel pooling and stacked pooling) is beneficial to capture a multiscale range to reduce computational cost. Rather than using the multicolumn approach (computationally complex), the concatenated-scale aggregation modules may increase counting accuracy. Moreover, the quality of the density map can be enhanced by using transposed convolutional layers at the cost of high complexity.*

#### 4.1.4. Multitask-CNN-CC Techniques

CNN-CC techniques that not only account for crowd counting but also for other tasks like classification, segmentation, uncertainty estimation, and crowd-behavior analysis are called multitask-CNN-CC techniques. We review the inter-relationship between these multiple tasks and their impact on the performance of individual tasks under the multitask-CNN-CC umbrella. Table 5 shows the detailed description of features, used datasets, and architecture of different algorithms under multitask-CNN-CC.

The authors in [92] proposed a ConvNet architecture to count the number of penguins. Due to occlusion and a scale-varying environment, a multitask learning technique was proposed to overcome foreground–background segmentation and uncertainty in density estimation. Idrees et al. [93] investigated the multitask technique by inter-relating three main problems: crowd counting, density estimation, and localization. In their work, the counting task was facilitated by density estimation and localization. Zhu et al. [94] proposed a deep and shallow FCN. Features extracted from a deep and shallow FCN were concatenated with the addition of two deconvolutional layers to make the output image similar to input image in terms of resolution. Instead of relying on modeling the visual properties, Huang et al. [95] proposed a semantic scene (body-structure-aware) CNN-based crowd-counting method. In their work, the crowd-counting problem was decomposed into a multitask problem. These multitasks involved the extraction of rich semantic-feature information, mapping the input scene image to the semantic scene model (body-part map and structured density map), and crowd counting. Yang et al. [96] proposed a multicolumn multitask neural network (MMCNN) to overcome drastic scale variation in an image. They used the multicolumn by incorporating three main changes. First, up- and downsampling was utilized to extract multiscale features. Second, deconvolution was used to account for loss due to downsampling. Third, loss per scale was minimized to make features more discriminative. Liu et al. [97] proposed a self-supervised method to increase the training data for enhancing accuracy. The ranked patches (cropped from original image) were used as side information. Moreover, multiscale sampling was utilized to further enhance accuracy.

**Remark** **3.** *First, training data can be increased by cropping an image into smaller patches (containing less than or an equal number of objects as compared to the larger patch). Second, the inter-relationship between different tasks may enhance counting accuracy. Third, deconvolution can be employed to enhance the quality of the density map. Finally, multiple tasks assist each other to increase the overall accuracy of the network. However, multitasking increases network complexity, which reduces its employability for real-time applications.*

### 4.2. Image-View-CNN-CC Techniques

The main focus of this category is to analyze an input image (arial or perspective) and accordingly design the network so that network accuracy can be improved. These techniques are very useful in medical imaging, monitoring of targeted areas through drones, and counting people through CCTV. Since camera angle, tilt, and position with respect to the object play a critical role in the development of any algorithm, we mainly divided image-view-CNN-CC into two subcategories: aerial-view-CNN-CC and Perspective-CNN-CC.

#### 4.2.1. aerial-View-CNN-CC Techniques

The set of techniques that mainly design the network according to input image (aerial-view-based) fall in this category. Techniques under this subcategory have applications in healthcare, commerce, the military, etc. Detailed limitations and characteristics of each of the algorithms under the umbrella of aerial-view-CNN-CC are given in Table 6.

In [98], the authors proposed a method to count the number of cells in a growing human embryo. They computed a bounding box by selecting a particular region (enclosing the embryo). Then, an end-to-end deep CNN was presented to count the number of cells in a microscopic image. Ribera et al. [99] proposed a regression model to estimate plants in an image (taken through a UAV). They minimized the number of neurons in the final layer to reduce the computational complexity of the network. However, issues like lack of large amounts of training data and occlusion were not addressed. The authors in [100] proposed a feature pyramid network with a VGG-style neural network for the segmentation and counting of microscopic cells. They utilized the downsampling of an image several times and learned features at varying scales to enhance segmentation and counting accuracy. However, downsampling affects the resolution of the ED map. Another approach was proposed by Xie et al. [101] to estimate the number of cells in a microscopic image. In this technique, two convolutional regression networks with a large receptive field (filters) were used to overcome cell clumps and the cell-overlapping problem.

**Remark** **4.** *By knowing the characteristics of the input image, techniques with less complexity and error rate can be designed. Individual regression models can be sequentially trained for low and high density to handle object clumps and sparsity. Occlusion can also be handled by feeding the downsampled patches into the CNN.*

#### 4.2.2. Perspective-CNN-CC Techniques

Techniques that mainly design the network according to the input image (perspective-view-based) fall in this category. These techniques are useful in varying-perspective scenarios with diverse scale variations. These techniques are applicable in dense-crowd-counting (e.g., sports events) scenarios having different perspectives, such as a shopping mall. By knowing the properties of the input image, techniques can be designed that have less complexity and high accuracy. The detailed features, used datasets, and architecture of each algorithm are shown in Table 7.

The authors in [102] proposed an adaptive CNN to incorporate perspective information. The convolutional-filter weights were adapted according to the current image scene by using perspective information. Zhao et al. [103] proposed a perspective-embedded deconvolution network (PE-CFCN-DCN) to model the varying size of pedestrians considering perspective distortion. They used a location-aware Gaussian function with varying kernel parameters for each annotated point (dot) to obtain the GTD. They also added a perspective map (one channel) as an additional channel to the RGB image (three channels) by modifying the filter depth from three to four channels. Perspective information was embedded with the deconvolution network (upsampling process) by utilizing structural information of different levels that help in the formation of a smooth and accurate density map (high-quality). Marsden et al. [104] proposed a multidomain patch-based (overlapped) regressor for object counting with the removal of redundant parameters in the model to reduce its complexity. A pretrained classification network was used to extract high-level features. The extracted features were mapped to the object count by using an FCNN. Further, switching among learned visual domains (people, wildlife, cells, and vehicles) could be accomplished with a subset of parameter interdomain sharing. This interdomain switching is very helpful in tackling different perspectives, scales, and density variations. Zhang et al. [105] proposed a switchable training method with multiobjective tasking. Two subtasks (estimating density and crowd count) influenced each other due to the introduction of a data-driven approach by choosing training scenes from all training data that have almost identical perspective maps with the target scene (test data). Shi et al. [106] proposed a perspective-aware CNN model where the perspective map was predicted and used as a perspective-aware weighting layer. This additional layer was responsible for combining thedensity maps obtained from varying-scale feature maps. The density and perspective maps were combined to provide the estimated count. The varying perspective and resolution problem was solved by Yao et al. [107] by proposing a Deep Spatial Regression Model (DSRM) using the CNN and LSTM. First, high-level features were extracted by using a CNN. Due to the high correlation among the overlapped patches, the LSTM structure used spatial information in adjacent regions to enhance counting accuracy. The final count was obtained by adding all the local patch counts.

*Remarks*: Perspective distortion may be reduced by inserting a perspective-aware weighting layer (separate layer) in the deconvolution network. Parameters among the different domains (trained on separate datasets) can be shared to overcome varying-perspective problems such as object-size and resolution variation.

### 4.3. T-CNN-NN Techniques

The set of techniques in this category are differentiated according to the approaches used to train the CNN, for example, training the CNN on the basis of a whole image or cropped patches. Such approaches can be used to improve the prediction accuracy of the network or the quality of its density map. Whole-image-based training minimizes the network computational cost at the cost of reduced accuracy, while patch-based training enhances network accuracy for high computational cost. These techniques are useful in medical imaging, commercial, and military applications. These techniques are categorized into patch-based-CNN-CC and whole-image-CNN-CC. Details are as follows.

#### 4.3.1. Patch-Based-CNN-CC Techniques

In these techniques, the CNN is trained by using cropped patches where a sliding window is run over the test image. These techniques are very useful in applications where there is enhanced resolution quality of the density map and it cannot be compromised, such as in cancer diagnosis. Both the affected cell count and the resolution of affected cells are important. The main objective of this category is to design a system for enhanced density-map quality at high computational cost. The detailed characteristics of each algorithm under patch-based-CNN-CC are shown in Table 8.

Cohen et al. [108] proposed a deep CNN inspired by inception networks. Instead of estimating the crowd count on the whole image, a smaller network is used to estimate the number of objects in a given receptive field. Overestimation of the crowd count in sparse areas by regression-based technique and underestimation of the crowd count in dense areas by detection-based techniques motivated the authors in [109] to proposed a detection and density-estimation (DecideNet) method that employed a counting mode based on density conditions. Inspired by the skip-connection method for crowd counting, the authors in [110] proposed an optimized method for information flow within different convolution and deconvolution layers. Convolution layers were used to detect the edges and colors, but this low-level information obtained from earlier layers may or may not have contributed to enhancing the performance of the network in terms of MAE. Therefore, a Gated U-Net (GU-Net) was employed to determine the amount of information passed to the final layer (convolution or fully connected) for a more accurate feature-selection process. Similar to the idea of [109], Xu et al. [111] proposed a depth-of-information-based guided crowd-counting method (Digcrowd) to deal with highly dense and varying-perspective images. Segmentation was performed on an image to divide it into two regions: far- and near-view regions. In the near-view region, people are counted by detection, and Digcrowd maps are used in the far-view region to map counted people to their density map. The authors in [112] used a head detector to find the varying size of a human head. After dividing images into multiple patches, an SVM classifier was used to classify crowded and noncrowded patches. In order to find the head size, regression was performed on each patch. After finding the head size, the total number of heads in a particular patch was calculated by dividing patch area with head size. The authors in [113] proposed a count-net technique by focusing on the head portion by filtering the background. Feature extraction and classification were also simultaneously performed with crowd counting. Zhang et al. [114] proposed a patch-based multicolumn CNN (MCNN) crowd-counting technique with a geometry-adaptive kernel for density estimation. The varying size of the receptive fields used in each CNN column was used to handle scale-varying objects (heads). However, the aggregation of the density map at the end may have decreased the quality of the ED map. Wang et al. [115] proposed a skip-connection CNN (SCNN) for crowd counting. The overall network used four multiscale units for extracting scale-varying features. Each multiscale unit consisted of three convolutional layers. Several multiscale units were used to extract the scale-varying features. Moreover, an augmentation strategy (without redundancy) was adopted by cropping the two patches (having different scales) from each input image. The CNN was individually trained on these two scales to overcome any drastic scale variations. Sam et al. [116] proposed a switch CNN technique by considering three regressors trained on low-, medium-, and high-density image patches. A switch (classifier) was used to direct the input patch to a particular regressor for addressing any density-variation issues.

**Remark** **5.** *Detection and regression can be sequentially employed on targeted image patches to enhance network prediction accuracy. Further, extracted low-level information about network edges and colors can be iteratively filtered to reduce the computational cost of the network.*

#### 4.3.2. Whole-Image-CNN-CC Techniques

Techniques in this subcategory perform whole-image-based inference, and are very useful in real-time applications due to the reduced computational cost. These techniques have applications in pedestrian counting, counting passing cars across CCTV, etc. The absence of negative sampling and lack of a data-driven approach are common in all the listed algorithms (see Table 9). Detailed characteristics of each algorithm under patch-based-CNN-CC are shown in Table 9.

A CNN-based fruit-counting technique was proposed by Rahnemoonfar et al. [117] by using a deep-simulated-learning algorithm. The network was trained on synthetic data (24,000 images consisting of variably sized tomatoes) with a whole-image-based training approach. A modified version of the Inception-ResNet architecture was used to implement the idea of fruit (tomatoes) counting. Sheng et al. [118] focused on the discriminative power of image representation by combining semantic information and locality-aware features (spatial and context information). By using the CNN, they mapped the pixel space into a semantic-feature map. Pixelwise features indicated a particular semantic class (e.g., road, person, pole, car, building). Furthermore, locality-aware features were used to exploit the local and contextual information. Later, the authors in [119] proposed a multiobjective technique by using residual deep-learning architecture (ResnetCrowd) to investigate crowd counting, violent-behavior detection, and density-level classification. The authors in [120] proposed a FCN for crowd counting by addressing the problems of scale variation and high density within an image. Instead of changing the receptive field (filter size) in a CNN, a scale-down version fed the network. To obtain the final count, they computed the mean of the downsampled images. Sindagi et al. [121] proposed a multitask cascaded CNN network to accurately learn crowd density and crowd classification. They exploited discriminative features (high-level prior) to handle high-level density variation within an image.

**Remark** **6.** *Counting accuracy could be enhanced by feeding the network with semantic and locality-aware features. High-level prior (i.e., density-level classification) with density estimation also take part in performance improvement.*

## 5. Applications of CNN-CC Algorithms

**Figure 6:**
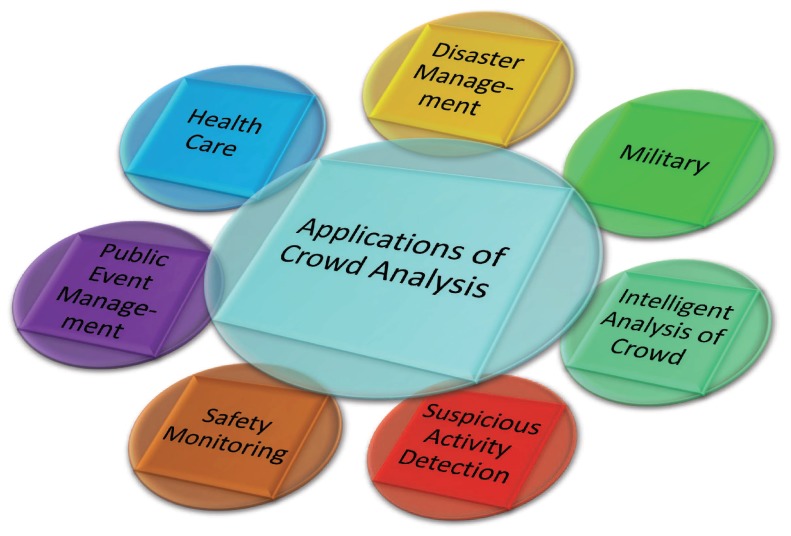
Applications of crowd analysis in different fields.

CNN-CC techniques have a diverse range of applications, as shown in Figure 6. These applications include intelligent crowd analysis, military applications, public-event management, disaster management, and health-care applications [23]. Detailed descriptions are given as follows.

*Intelligent Crowd Analysis*: Crowd-counting techniques can be employed to gather information for intelligent analysis and inference. For example, the queue length in front of a billing reception center (electric, gas, and water bills), especially in developing countries, could be observed and analyzed to accordingly optimize the number of staff members. Traffic-signal wait time could be optimized with respect to crowd flow, especially during office hours. Moreover, appropriate product placement can be done in big malls and stores according to the interest of people [9,122,123].

*Military Applications*: CNN-CC techniques can be used in military applications such as counting the number of moving drones or fighter jets or the number of enemy soldiers and their weapons. Thus, the strength of the enemy’s armed forces could be estimated to counter a surge [52,124,125].

*Public-Event Management*: CNN-CC techniques can be used in concerts, sports events, and political rallies to count the number of people. Thus, these events can be managed by analyzing and counting the crowd to avoid disastrous situations. This would also be beneficial in properly managing available resources, such as spatial capacity and optimizing crowd movements [126,127,128].

*Disaster Management*: There are different overcrowding scenarios, like musical concerts and religious gatherings, which could be life-threatening when a portion of the crowd panics and charges in random directions. In the recent past, huge numbers of people have died from suffocation in highly crowded areas in different public-gathering events. Early detection of overcrowding and better crowd management in political rallies, sports events, and musical concerts can be made possible by analyzing the crowd gathering [129,130,131].

*Suspicious-Activity Detection*: Terror attacks in public places can be minimized by using crowd analysis and violent-crowd-behavior detection techniques. Traditional handcrafted methods do not perform well in harsh and densely crowded events, and could be replaced by CNN-based face recognition and detection techniques for better crowd analysis [132,133,134,135].

*Health-Care Applications*: CNN-CC techniques play an important role in health-care systems, especially with patients suffering from cancer and other diseases where it is important to count a number of cancerous cells for early-stage diagnosis. The authors in [136] proposed a deep model for cell detection in zebrafish images. This framework was used to detect tyrosine hydroxylase cells in zebrafish brain images. Further, the authors in [137] presented a CNN-based model for histopathologic cancer diagnosis through a deep-learning architecture to increase the objectivity and efficiency of histopathology-slide analysis. The authors in [138] also diagnosed skin cancer by using skin images with a deep NN. Finally, the authors in [139] trained a deep NN to predict different liver diseases.

*Safety Monitoring*: A huge number of CCTV monitoring systems at airports, religious gathering places, and public locations enable easier crowd monitoring. CNN-CC algorithms could be further analyzed to detect behaviors and congestion-time slots to ensure the safety and security of the public [140]. For example, the authors in [141] presented a multicamera approach to detect dangers by analyzing crowd density. In other works, such as [142,143], the authors proposed a surveillance system to generate a graphical report by analyzing crowds and their flow in various directions through CCTV cameras.

## 6. Three-Dimensional Crowd Counting

The widespread usage of CCTV monitoring systems at airports, religious gathering places, and public places enable easier monitoring of crowd. However, traditional crowd-counting methods with classification [144,145] and segmentation [146] via deep-learning techniques rely on 2D datasets instead of video crowd counting. The task of crowd counting from videos is challenging due to severe occlusions, scene-perspective distortions, diverse crowd distributions, and especially complex network architectures. Limitations in terms of complex networks (high computational cost) restrict researchers from deploying real-time crowd-counting algorithms. For that, we need to simplify deep-learning models so that they can be easily deployed. Rapidly growing crowd-counting technologies demand investigations to reduce NN computational cost and network complexity. More specifically, the reduction of complex models to simpler ones [147,148] encourages the wide adoption of such models in remote stations for real-time applications, such as crowd analysis in autonomous vehicles.

Dimensionality reduction is used to reduce the complexity of machine-learning networks and reduce overfitting. The authors in [149] proposed a principal-component-analysis (PCA)-based nonparametric, unsupervised technique for dimensionality reduction. The authors in [150] investigated PCA applications, kernel PCA (KPCA), and independent component analysis (ICA) with an SVM for feature extraction. PCA was used to linearly transform the original inputs into uncorrelated new features, whereas the linearly transformed features in ICA are statistically independent. KPCA is nonlinear PCA that is done by generalizing the kernel method into linear PCA. Similarly, the authors in [151] proposed an unsupervised method for dimensionality reduction called Locally Linear Embedding (LLE). By maintaining the geometric features of a nonlinear feature structure, it reduces the n-dimension feature space. LLE optimization does not involve local minima by mapping inputs into a single coordinate having lower dimensions. By observing the performance of model simplifications in machine-learning approaches, different authors also proposed simplified models for deep learning [152,153,154,155].

## 7. Performance Evaluation of CNN-CC Algorithms

In this section, our main goal was to evaluate the selected existing CNN-CC algorithms. For evaluation purposes, we considered a common performance metric: MAE, where *N* is the number of test samples, $y_{i}$ is used for ground-truth count, and $y_{i}^{\prime }$ is the estimated count of *i* th sample.

(1) $$MAE=\frac{1}{N}\sum_{i=1}^{N}\left|y_{i}-y_{i}^{\prime }\right|$$

For comparison, we chose the following benchmark techniques:

- Refs. [73,74,75] as Basic-CNN-CC algorithms tested via the USCD and UCF datasets.
- Refs. [77,78,79,80,81] as Context-CNN-CC algorithms tested via the UCF, ShanghaiTech-A (STA) and ShanghaiTech-B (STB) datasets.
- Refs. [83,84,85,86,87,88,89,90,91] as Scale-CNN-CC algorithms tested via the UCF, STA and STB datasets.
- Refs. [94,95,96,97] as Multi-task-CNN-CC algorithms tested via the UCF, STA and STB datasets.
- Refs. [102,104,105,106,107] as Perspective-CNN-CC algorithms tested via the UCF, STA and STB datasets.
- Refs. [109,110,111,112,113,114,115,116] as Patch-based-CNN-CC algorithms tested via the UCF, STA and STB datasets.
- Refs. [119,120,121] as whole-image-CNN-CC algorithms tested via the UCF, STA and STB datasets.

**Figure 7:**
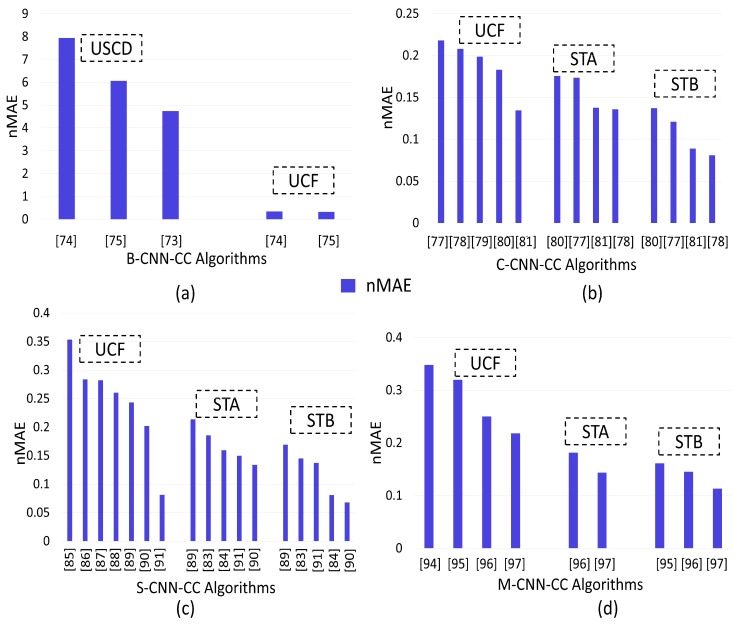
Normalized Mean Absolute Error (nMAE) of network-CNN-CC algorithms tested on different datasets: (**a**) basic-CNN-CC, (**b**) context-CNN-CC, (**c**) scale-CNN-CC, and (**d**) multitask-CNN-CC.

Figure 7a shows that the normalized MAE (nMAE) of [74] was relatively higher than that of [75] when tested on the USCD dataset. This is because of an underestimation of layer boosting that iteratively increased the number of network layers due to selective sampling. Further, the nMAE of [73] was relatively less than that of [75] when tested on the USCD dataset. This is because the two subproblems (crowd-velocity and -density estimation) in [73] assisted each other to enhance performance. Similarly, the nMAE of [75] was relatively less than that of [74] when tested on the UCF dataset due to previously mentioned reasons. Hence, we concluded that, instead of the direct insertion of new layers in the CNN, iteratively increasing the number of layers in a trained network may improve system performance in terms of nMAE. System performance may further be improved if a multitasking approach is employed.

Figure 7b shows that the nMAE of [81] was relatively lower than that of [77,78,79,80] when tested on the UCF dataset. The reason was the consideration of a spatial transformer network (to tackle scale and rotation), and a local refinement network (to account for contextual information) in [81]. Further, the nMAE of [78] was relatively lower than that of [77,80,81] when tested on the STA and STB datasets. This is due to the consideration of dilated convolution by expanding the kernel that is useful in extracting contextual information. By comparing the nMAE, the performance of [78] was the relative lowest from all compared algorithms when tested on the STB dataset due to the tilted behavior of STB towards low density. Hence, after observing the performance of context-CNN-CC, we concluded that counting accuracy could be enhanced on datasets with diverse scenes and varying densities by solving pose variations and photographic angles for accurate density estimation. Performance could also be increased on low-density datasets by adopting dilated convolution.

Figure 7c depicts that the nMAE of [91] was relatively low when compared to that of [85,86,87,88,89,90] on the UCF dataset. This is due to the introduction of a novel ASCP framework (inspired from GANs). Adversarial loss instead of $l_{2}$ regression loss also enhanced accuracy. Further, the nMAE of [90] was relatively low when compared to that of [83,84,89,91] on the STA and STB datasets. This is because the combination of a scale-aware network with transpose convolution enhanced the counting accuracy and quality of the density map. Further, the nMAE of [91] was relatively low when compared to that of [84] on the STA dataset due to the above-mentioned reasons. However, [91] had a relatively high nMAE as compared to that of [84] on the STB dataset. This is due to the consideration of scale-aggregation modules with a combination of Euclidian and local-pattern consistency loss by [84]. Hence, we concluded that there were two main scale-variation issues that need solutions: (1) A scale-specific network performs poorly on unknown scales, which results in low-quality density maps. (2) The coherence issue among different density maps is not properly addressed (summing the individual local counts may not be necessary to approximate the total count).

Figure 7d depicts that the nMAE of [97] was relatively low when compared to that of [94,95,96] on the UCF dataset. The reason was the consideration of a self-supervised learning technique (increased training-data size). Further, the nMAE of [97] was relatively low when compared to that of [95,96] on the STA and STB datasets due to the above-mentioned reasons. Further, [96] had a relatively low nMAE when compared to that of [94,95] on the UCF and STB datasets. This was due to handling scale variation by using multikernels (parallel) with a multitask approach. Hence, we concluded that counting accuracy could be enhanced by focusing on calculating the accurate GTD, and increasing the number of training data improves ED quality. Drastic scale variation could also be handled by using the combination of semantic information (body-part information) with up- and downsampling.

**Figure 8:**
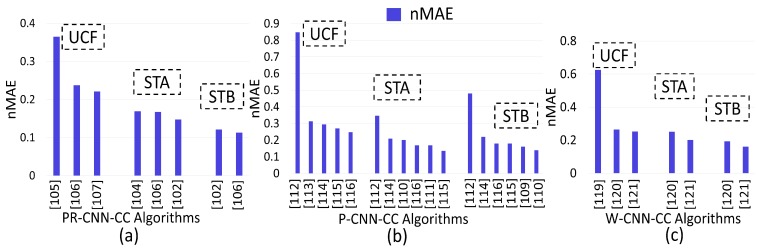
nMAE of CNN-CC algorithms tested on different datasets: (**a**) perspective-CNN-CC, (**b**) patch-based-CNN-CC, and (**c**) whole-image-CNN-CC.

Figure 8a shows that the nMAE of [107] was relatively low when compared to that of [105] and [106]. This is because Yao et al. [107] used a combination of CNN (extracting high-level information) and LSTM (using spatial information to regress the local count from adjacent patches) to increase network prediction accuracy. Further, the nMAE of [102] was relatively low when compared to that of [104,106] on the STA dataset. This is due to the incorporation of side (contextual) information having perspective weights in the CNN. However, the nMAE of [106] was relatively low when compared to that of [102] with a low margin on the STB dataset. The accuracy enhancement was due to separate perspective-aware layers considered by [106]. Hence, we concluded that, by combining the fine-tuning part (retrieving training scenes from all training datasets that had a similar perspective map with the target scene) with a deconvolution network increases accuracy and enhances ED map quality.

Figure 8b depicts that the nMAE of [116] was relatively low when compared to that of [112,113,114,115] on the UCF dataset. This is due to the consideration of density-level classification of image patches with a density-oriented-based regressor approach. Further, the nMAE of [115] was relatively low when compared to that of [110,111,112,114,116] on the STA dataset. This is due to consideration of a skip connection with scale-oriented training to handle varying-scale issues. The nMAE of [110], on the other hand, was relatively low when compared to that of [109,112,114,115,116] on the STB dataset. This was due to the consideration of controlled flow of information through the convolution and deconvolution layers in [110]. We therefore conclude that for datasets with a dense and diverse range of densities, a specific-task-oriented regressor and deconvolution increase accuracy for estimating a high-quality density map. However, low-density datasets can be tackled by using a patch-based augmentation (varying-scale) strategy, and optimized information flow within the convolution and deconvolution layers by addressing the scale-varying issue caused by the perspective view.

Figure 8c shows that the nMAE of [121] was relatively low when compared to that [119,120] on the UCF dataset. The reason of this error reduction was the consideration of the high-level prior with density estimation. Further, the method in [120] has a low nMAE when compared to that of [119] on the UCF dataset. This is due to addressing the problem of dense crowds by feeding images at multiple scales, as in Marsden et al. [120]. Further, the nMAE of [121] was relatively low when compared to that of [120] on the STA and STB datasets due to the above-mentioned reasons. Hence, we conclude that high-crowd-density issues can be solved up to an extent by varying the image scale. Multitasking makes the system more complex for real-time applications.

By comparing the performance of subcategories of Network-CNN-CC, we concluded that density-estimation accuracy is increased by using adversarial loss instead of regression loss. The quality of the density map is also enhanced by using GANs, as per Shen et al. [91]. The work in [91] had the lowest nMAE as compared to the rest of the algorithms under network-CNN-CC when tested on the most challenging UCF dataset. The enhanced performance was proved by [90] on the STA and STB datasets under network-CNN-CC. This is due to consideration of training loss with a scale-aware network by using transpose convolution. Similarly, by observing the performance of subcategories of image-view-CNN-CC, we concluded that [102,106,107] performed well on the STA, STB, and UCF datasets. This is due to the utilization of a CNN with LSTM for spatial information to regress the local object count in adjacent regions (patches) in [107]. The enhanced performance of [84,106] was due to consideration of the perspective information and perspective-aware weighting layer. By investigating the training-CNN-CC, we concluded that [110,115,116] performed well on the STB, STA, and UCF datasets, respectively. This enhanced performance was due to using a density-level classifier with a density-oriented regressor in [116]. The reason for the high performance was the usage of a skip connection with scale-oriented training in [115]. The algorithm in [110] performed well due to optimized information movement within the convolution and deconvolution layers. Finally, we concluded that the algorithm of Shen et al. [91] performed well on the UCF dataset, while that of Cao et al. [90] showed better performance on the STA and STB datasets.

## 8. Conclusions and Key Observations

Intelligent crowd counting and its analysis are a future development of traditional handcrafted methods. By leveraging the tight integration of machine-learning and artificial-intelligence technologies with traditional crowd-counting techniques, intelligent crowd counting and its analysis provide advanced features such as adaptive control for dynamic crowd gatherings, and their wide-area monitoring/surveillance. These advanced features can improve many crowd-management-related tasks in terms of efficiency, capacity, reliability, and safety. CNN-CC techniques can effectively support many applications that require adaptive monitoring, identification, and management over diverse crowd-gathering horizons. In this article, we presented a comprehensive review of CNN-CC and density-estimation techniques. We mainly categorized CNN-CC techniques into network-, image-view-, and training-CNN-CC. Moreover, we subcategorized the three main categories and accordingly summarized recent research articles. In each subcategory, we discussed the latest research articles in terms of their key features, used datasets, and architectures. We also critically reviewed the research articles in terms of key characteristics and deficiencies. Finally, we provided quantitative comparison results of the sub- and main categories to facilitate future researchers. On the basis of our comprehensive review, we conclude the following key observations.

- Counting accuracy of basic-CNN-CC is enhanced by removing redundant samples, while multitasking improves the overall accuracy of an algorithm.
- The quality of a density map in context-CNN-CC is enhanced by using a deeper dilated CNN, while counting accuracy is enhanced by using an adaptive-density network through pose-variation-based solutions.
- By investigating scale-CNN-CC, we observed that counting accuracy is improved by using stacked pooling that reduces computational cost. Moreover, concatenated-scale aggregation modules increase accuracy, and the quality of the density map is enhanced.
- Counting the accuracy of multitask-CNN-CC is increased by using self-supervised learning, inter-relations between multiple tasks, and up- and downsampling. However, multitasking makes the system more complex for real-time applications. Density-map quality is also enhanced by using deconvolution layers.
- Performance i of aerial-view-CNN-CC n terms of nMAE is increased by using multiple regression models, and occlusion is handled by feeding the downsampled patches in the CNN.
- Counting accuracy of the PRCC is enhanced by inserting a perspective-aware layer in the deconvolution network, parameter sharing within different domains, and retrieving training scenes from all training datasets that have similar perspective maps with target scenes.
- The nMAE of patch-based-CNN-CC is increased by using detection and regression depending on image density and the optimal transfer of information within CNN layers. For dense datasets, the combination of density-level classification, a specific task-oriented regressor, and deconvolution increase accuracy with the estimation of high-quality density maps. Density datasets are tackled by using a patch-based augmentation (varying scale) strategy.
- The counting accuracy of whole-image-CNN-CC is improved by exploiting semantic and locality-aware features, and density-level classification. Diverse-crowd-density issues are also fixed to some extent by varying image scales, making these techniques highly applicable in real-time applications.

For future work, we will integrate Restricted Boltzmann Machines (RBMs) into a CNN-based crowd-counting network. Further, we will enhance the accuracy and quality of estimated density maps by using varying receptive fields. Besides accuracy, we are interested in reducing the computational cost (number of parameters) of CNN-based crowd-counting networks.

## Figures and Tables

**Table 1:** Summary of different crowd-counting datasets with their intrinsic features.

Dataets	USCD [17]	Mall [4]	UCF [69]	WE [28]	STA [26]	STB [26]
**No. of images (NOI)**	2000	2000	50	3980	482	716
**Resolution**	158 × 238	320 × 240	Varied	576 × 720	Varied	768 × 1024
**Minimum head count**	11	13	94	1	33	9
**Average head count**	25	-	1279	50	501	123
**Maximum head count**	46	53	4543	253	3139	578
**Total head count (THC)**	49,885	62,325	63,974	199,923	241,677	88,488
**Qualitative features**	Collected from video camera, ground-truth annotation, low-density dataset, no perspective variation	Collected from surveillance camera, diverse illumination condition; compared to USCD, it has higher density, no scene-perspective variations	Collected from various places like concerts, marathons, diverse scenes with wide range of densities, challenging datasets as compared to USCD and Mall	Specific for cross-scene crowd-counting large diversity, but limited as compared to UCF, not dense as compared to UCF, more images	Chosen from Internet, large scale, largest in terms of number of annotated people, large density as compared to (B), diverse scenes, and varying densities	Collected from Shanghai, varying scale and perspective, nonuniform density level in many images, making it tilt towards the low-density level

**Table 2:** Summary of advantages and limitations of basic-CNN-CC algorithms.

Technique	Features	Datasets	Negative Samples		Data Driven		Architecture
			Yes	No	Yes	No	
Fu et al. [70]	Real-time approach	PETS_2009, Subway video, Chunix_Road video		✓		✓	ConvNets
Mundhenk et al. [71]	Contextual information, creation of large datasets of cars	Cars Overhead with Context (COWC),	✓			✓	AlexNet, Inception
Wang et al. [72]	End-to-end deep CNN regression model	UCF	✓		✓		FCN
Zhao et al. [73]	Joint learning of crowd density and velocity	USCD, [LHI, TS, CNN] *		✓		✓	FlowNet
Hu et al. [74]	Two supervisory signals: crowd count and crowd density	UCF, USCD		✓		✓	ConvNets
Walach et al. [75]	Gradient boosting and selective sampling, and elimination of low-quality training samples	UCF, USCD, [Bacterial Cell, Make 3D] *	✓		✓		Boosting Net

* Private datasets.

**Table 3:** Summary of advantages and limitations of Context-CNN-CC algorithms.

Technique	Features	Datasets	Negative Samples		Data Driven		Architecture
			Yes	No	Yes	No	
Chattopadhyay et al. [76]	Associative subitizing	PASCAL VOC, COCO					ConvNet
Zhang et al. [77]	Attention model for head detection	UCF, STA, STB					AM-CNN
Li et al. [78]	Dilated convolution and multiscale contextual information	UCF, STA, STB, WE					CSRNet
Han et al. [79]	Combination of correlation and MRF	UCF					ResNet
Wang et al. [80]	Density adaption network	ST, UCF					DAN, LCN, HCN
Liu et al. [81]	Spatially aware network	ST, UCF, WE					Local Refinement Network

**Table 4:** Summary of advantages and limitations of scale-CNN-CC algorithms.

Technique	Features	Datasets	Negative Samples		Data Driven		Architecture
			Yes	No	Yes	No	
Liu et al. [82]	Geometry-aware crowd counting	ST, WE, Venice		✓		✓	Siamese
Huang et al. [83]	Exploits cross-scale similarity	ST, WE		✓		✓	Wide and Deep
Kang et al. [84]	Image pyramid to deal with scale variation	ST, WE, USCD		✓		✓	VGG network
Boominathan et al. [85]	Combination of deep and shallow networks	UCF		✓		✓	VGG-16
Zeng et al. [86]	Single multiscale column	ST, UCF		✓		✓	Inception
Kumagai et al. [87]	Integration of multiple CNNs (gating and expert CNN)	UCF, Mall		✓		✓	MoC-CNN
Onoro-Rubio et al. [88]	CCNN for mapping the appearance of image patch to its density map; Hydra CNN is scale-aware model	UCF, USCD, TRANCOS		✓		✓	CCNN, Hydra
Shi et al. [89]	Dynamic data-augmentation strategy, NetVLAD	ST, UCF, WE		✓		✓	VGG-like net
Cao et al. [90]	Multi-scale feature extraction with scale aggregation modules	UCF, STA, STB, USCD		✓		✓	SANet
Shen et al. [91]	GANs-based network, novel regularizer	ST, UCF, USCD		✓		✓	ACSCP

**Table 5:** Summary of advantages and limitations of multitask-CNN-CC algorithms.

Technique	Features	Datasets	Negative Samples		Data Driven		Architecture
			Yes	No	Yes	No	
Arteta et al. [92]	Multitasking: foreground and background segmentation, uncertainty, and density estimation	Penguins dataset	✓		✓		ConvNet
Idrees et al. [93]	Multitasking with loss optimization	UCF-QNRF		✓		✓	DenseNet
Zhu et al. [94]	Combination of pedestrian flow statistics task with people counting	UCF, [DH302IMG, DH302VID] *		✓		✓	VGGNet-16
Huang et al. [95]	Body structure-aware methods	STB, UCF, USCD		✓	✓		Multi-column body-part aware model
Yang et al. [96]	Multicolumn multitask CNN focusing on drastic scale variation	ST, UCF, USCD, MALL, WE		✓		✓	MMCNN
Liu et al. [97]	Self-supervised tasking	UCF, STA, STB		✓	✓		VGG-16

* Private Datasets.

**Table 6:** Summary of advantages and limitations of aerial-view-CNN-CC algorithms.

Technique	Features	Datasets *	Negative Samples		Data Driven		Architecture
			Yes	No	Yes	No	
Khan et al. [98]	Automatic approach to select a region of interest by computing a bounding box that encloses the embryo	Time-lapse image sequences		✓	✓		Architecture of Krizhevsky
Ribera et al. [99]	Plants are estimated by using the regression model instead of classification	RGB UAV images of sorghum plants		✓		✓	Inception-v2
Hernnandez et al. [100]	Feature pyramid network	BBBC005		✓		✓	VGG-Style NN
Xie et al. [101]	Two convolutional regression networks	RPE, T and LBL cells		✓		✓	VGG-net

* Private Datasets.

**Table 7:** Summary of advantages and limitations of Perspective-CNN-CC algorithms.

Technique	Features	Datasets	Negative Samples		Data Driven		Architecture
			Yes	No	Yes	No	
Kang et al. [102]	Incorporating side information (perspective weights) in CNN by using adaptive convolutional layers	USCD		✓		✓	ACNN
Zhao et al. [103]	Perspective embedded deconvolution network	WE		✓		✓	PE-CFCN-DCN
Marsden et al. [104]	Multidomain patch-based regressor	ST, Penguin, Dublin cell *		✓		✓	VGG16
Zhang et al. [105]	Cross scene crowd counting, human body shape and perspective variation are considered	UCF		✓	✓		Crowd CNN model
Shi et al. [106]	Perspective-aware weighting layer	UCF, WE, STA, STB		✓		✓	PACNN
Yao et al. [107]	General model based on CNN and LSTM	ST, UCF, WE		✓	✓		DSRM with ResNet

* Private Datasets.

**Table 8:** Summary of advantages and limitations of patch-based-CNN-CC algorithms.

Technique	Features	Datasets	Negative Samples		Data Driven		Architecture
			Yes	No	Yes	No	
Cohen et al. [108]	Smaller network used for estimation in given receptive field	[VGG, MBM] *		✓		✓	Count-ception
Liu et al. [109]	Detection and density-estimation network	Mall, STB, WE		✓		✓	DecideNet
Onro-Rubio et al. [110]	Joint feature extraction and pixelwise object density	ST, USCD, TRANSCOS		✓		✓	GU-Net
Xu et al. [111]	Depth-information-based method	STB, Mall, ZZU-CIISR		✓	✓		Multi-scale network
Shami et al. [112]	Head-detector-based crowd-estimation method	ST, UCF	✓			✓	ImagNet
Zhang et al. [113]	Aggregated framework	UCF, AHU-CROWD	✓			✓	count-net
Zhang et al. [114]	Multicolumn CNN with varying receptive fields	ST, UCF		✓	✓		MCNN
Wang et al. [115]	Skip-connection CNN with scale-related training	ST, UCF		✓	✓		SCNN
Sam et al. [116]	Switch CNN multidomain patch-based regressor	ST, UCF, WE		✓	✓		Switch CNN

* Private Datasets.

**Table 9:** Summary of advantages and limitations of whole-image-CNN-CC algorithms.

Technique	Features	Datasets	Negative Samples		Data Driven		Architecture
			Yes	No	Yes	No	
Rahnmonfar et al. [117]	Simulated learning, and synthetic data for training, tested on real images	Fruit dataset *		✓		✓	Inception-ResNet
Sheng et al. [118]	Pixel-level semantic-feature map, learning locality-aware features	USCD, Mall		✓		✓	Semantic-feature map and W-VLAD encoding
Marsden et al. [119]	Simultaneous multiobjective method for violent-behavior detection, crowd counting and density-level classification, creation of new dataset	UCF		✓		✓	ResNetCrowd
Marsden et al. [120]	Multiscale averaging to handle scale variation	ST, UCF		✓		✓	FCN
Sindagi et al. [121]	Multitask end-to-end cascaded network of CNNs to learn both crowd-count classification and density estimation	ST, UCF		✓		✓	Cascaded network

* Private Datasets.

## Acronyms

NNs	Neural Networks
CNNs	Convolutional NNs
RNNs	Recurrent NNs
FCL	Fully Connected Layer
UAV	Unmanned Aerial Vehicle
ReLU	Rectified Linear Unit
GTD	Ground Truth Density
ED	Estimated Density
GLCM	Gray Level Co-Occurrence Metrics
HOG	Histogram Oriented Gradient
LBP	Local Binary Pattern
KLT	Kanade–Lucas–Tomasi
GANs	Generative Adversarial Networks
MAE	Mean Absolute Error
MSE	Mean Square Error
STA	ShanghaiTech-A (a dataset)
STB	ShanghaiTech-B (a dataset)
WE	World Expo 10 (a dataset)
CNN-CC	CNN Crowd Counting
Network-CNN-CC	Network-based CNN-CC techniques
Basic-CNN-CC	Basic CNN-CC techniques
Context-CNN-CC	Context-aware CNN-CC techniques
Scale-CNN-CC	Scale-aware CNN-CC techniques
Multi-task-CNN-CC	Multitask CNN-CC techniques
Image-view-CNN-CC	Image-view-based CNN-CC techniques
Aerial-view-CNN-CC	Aerial-view-based CNN-CC techniques
Perspective-CNN-CC	Perspective-view-based CNN-CC techniques
Patch-based-CNN-CC	Patch-based CNN-CC techniques
Whole-image-CNN-CC	Whole-image-based CNN-CC techniques
Training-CNN-CC	Training-approach-based CNN-CC techniques
